# Responses of *Microcystis* Colonies of Different Sizes to Hydrogen Peroxide Stress

**DOI:** 10.3390/toxins9100306

**Published:** 2017-09-27

**Authors:** Mixue Liu, Xiaoli Shi, Chao Chen, Li Yu, Chuang Sun

**Affiliations:** 1 State Key Laboratory of Lake Science and Environment, Nanjing Institute of Geography and Limnology, Chinese Academy of Sciences, Nanjing 210008, China; liumixue15@mails.ucas.ac.cn (M.L.); chaochenhc@163.com (C.C.); yuli514605@163.com (L.Y.); 2 College of Resources and Environment, University of Chinese Academy of Sciences, Beijing 100049, China; 3 College of Environmental Science and Engineering, China West Normal University, Nanchong 637009, China; 4 College of Animal Science and Technology, Anhui Agriculture University, Hefei 230036, China; bestornothing0330@163.com

**Keywords:** hydrogen peroxide, *Microcystis*, colony size, antioxidative response

## Abstract

*Microcystis* blooms have become a ubiquitous phenomenon in freshwater ecosystems, and the size of *Microcystis* colonies varies widely throughout the year. In the present study, hydrogen peroxide (H_2_O_2_) was applied to test the effect of this algaecide on *Microcystis* colonies of different sizes and to evaluate the colonies’ antioxidant strategy. The results showed that *Microcystis* populations collapsed under treatment with 5 mg/L H_2_O_2_ at colony sizes smaller than 25 μm. A dosage of 20 mg/L H_2_O_2_ was necessary to efficiently control *Microcystis* colonies larger than 25 μm. The enzymatic and non-enzymatic antioxidant systems of different colonies exhibited various strategies to mitigate oxidative stress. In small colonies, superoxide dismutase (SOD) activity was readily stimulated and operated with catalase (CAT) activity to eliminate reactive oxygen species (ROS). In colonies larger than 25 μm, the antioxidant enzyme CAT and antioxidant substance glutathione (GSH) played major roles in mitigating oxidative stress at H_2_O_2_ concentrations below 20 mg/L. In addition, application of the algaecide led to the release of intracellular-microcystins (MCs), and oxidatively-driven MCs reached high concentrations when colony size was larger than 100 μm. Algaecide control measures should be implemented before the formation of large colonies to limit the algaecide dosage and MC release.

## 1. Introduction

The frequent outbreak of freshwater cyanobacteria blooms has become a ubiquitous phenomenon throughout the world, and outbreaks have been increasing in frequency over the past several decades [1]. Cyanobacteria bloom formation is thought to be controlled by several physical, chemical and biological factors, including temperature, light conditions, water currents, nutrient availability, the intrinsic physiological characteristics of cyanobacteria, and other organisms [2,3]. Cyanobacteria have the potential to produce health-affecting toxins and odorous compounds that restrict the usage of lakes and lake water for ecosystem services of societal and economic importance, including the provision of drinking water, irrigation, aquaculture, fish breeding, and recreation. Water blooms can lead to severe water quality problems and negatively affect fisheries as well as civil, industrial and recreational uses of water resources [4]. *Microcystis* is one of the dominant genus in cyanobacteria blooms [3]. This species produces hepatotoxin microcystins (MCs), which are monocyclic heptapeptides that act as eukaryotic protein phosphatase inhibitors and tumor promoters. Microcystins have the common structure cyclo (D-alanine-L-X-d-erythro-β-methylaspartic acid-L-Y-Adda-d-glutamate-N-methyldehydroalanine), where X and Y are variable L-amino-acids. So far, more than 100 structurally different MC have been found [5]. However, MC-LR, MC-YR, MC-RR (where L = leucine, R = rginine, Y = tyrosine) are the most common and abundant MCs present in diverse water systems [6]. Microcystins cause acute or chronic poisoning in humans and livestock as well as in various cyanobacterial grazers [7]. Consequently, effective measures are urgently required to prevent and control algal blooms. Establishing decreased nutrient levels and restricting further nutrient inputs are together considered to be a fundamental approach for eradicating cyanobacteria blooms [7]. However, this approach is time consuming and frequently economically unfeasible in Lake Taihu [8].

Measures to control water blooms include physical, biological and chemical measures. As a chemical algaecide, hydrogen peroxide has received considerable attention for its benefits, including low eco-toxicological impacts (rapid degradation into oxygen and water), selective inhibition of target cyanobacterium *Microcystis* from mixed phytoplankton communities and enhancement of the oxidative degradation of MCs [9,10]. The effectiveness of H_2_O_2_ in inhibiting algae depends greatly on light intensity, algae species and colony size [10,11,12,13]. Several studies have shown that algal colonies of different sizes vary in their sensitivity to some exogenous substances, with small colonies being more sensitive than the large ones. For example, small colonies of *Microcystis* have a lower resistance to copper than do the large colonial *Microcystis* [14]. The colony size of *Microcystis* commonly increases with the development of cyanobacterial blooms, with the mean number of cells per colony increasing from one to hundreds or more. Small colonies tend to dominate in the winter, and large colonies typically form and dominate in the summer [3,15]. To our knowledge, previous research has mainly focused on the inhibition effectiveness of H_2_O_2_ on mixed algal communities/blooms in the field or on isolated species in the laboratory [12,16,17]. It is important to understand how *Microcystis* colonies of different sizes respond to H_2_O_2_ stress to determine the appropriate H_2_O_2_ dosages for efficiently mitigating cyanobacterial blooms at different growth stages.

As reported in previous studies, the addition of H_2_O_2_ can generate oxidative stress and trigger cellular antioxidant defense system responses in algae. Under certain oxidative conditions, algae can survive by increasing their antioxidant defenses, including the production of enzymatic antioxidants and non-enzymatic antioxidants. Among the enzymatic antioxidants, superoxide dismutase (SOD) converts the superoxide anion (O_2_^−^) to hydrogen peroxide (H_2_O_2_), and catalase (CAT) is involved in directly eliminating H_2_O_2_. Reduced glutathione (GSH) is a crucial non-enzymatic antioxidant that scavenges H_2_O_2_ [18]. However, when the oxidative stress caused by H_2_O_2_ exceeds a certain threshold, the enzymatic and non-enzymatic antioxidant systems become increasingly ineffective. Such conditions lead to the inhibition of various physiological processes, including photosynthesis, synthesis of pigments and circadian rhythms, and ultimately lead to cell death [13,16]. In addition, cell membranes made of unsaturated phospholipids are vulnerable to oxygen radical attack, and damage can result in accumulation of malondialdehyde (MDA) [19]. Therefore, increased MDA content is a vital sign of cellular oxidative damage.

The release of microcystins (MCs) following the addition of a chemical algaecide is a large concern. Microcystin within phytoplankton cells and microcystin released into the surrounding water (dissolved) are referred to as intracellular- and extracellular-MCs, respectively. Previous studies have demonstrated that several chemical algaecides cause cell lysis, which increases the concentration of extracellular-MCs. These algaecides include copper sulfate, hydrogen peroxide, diuron and ethyl 2-methyl acetoacetate [20,21]. Moreover, it has been reported that extracellular- MCs are difficult to remove by using traditional water treatment technologies [22]. Therefore, the prevention or limitation of extracellular-MCs release is critically important for algaecide use in combating cyanobacterial blooms.

In the present study, a *Microcystis* population was sampled and fractionated according to colony size, and colonies of different sizes were exposed to different H_2_O_2_ concentrations in the laboratory. Chlorophyll-a content, photosynthetic capacity, esterase activity, antioxidant response, and H_2_O_2_, extracellular-MCs and intracellular-MCs concentrations were analyzed. The objectives of this study were to (i) assess how cyanobacteria colonies of different sizes respond to H_2_O_2_ oxidative stress, (ii) investigate the strategies to combat oxidative stress among cyanobacteria colonies of different sizes, and (iii) monitor and evaluate MC release under H_2_O_2_ treatment in cyanobacteria colonies of different sizes.

## 2. Results

### 2.1. Degradation of H_2_O_2_

**Figure 1:**
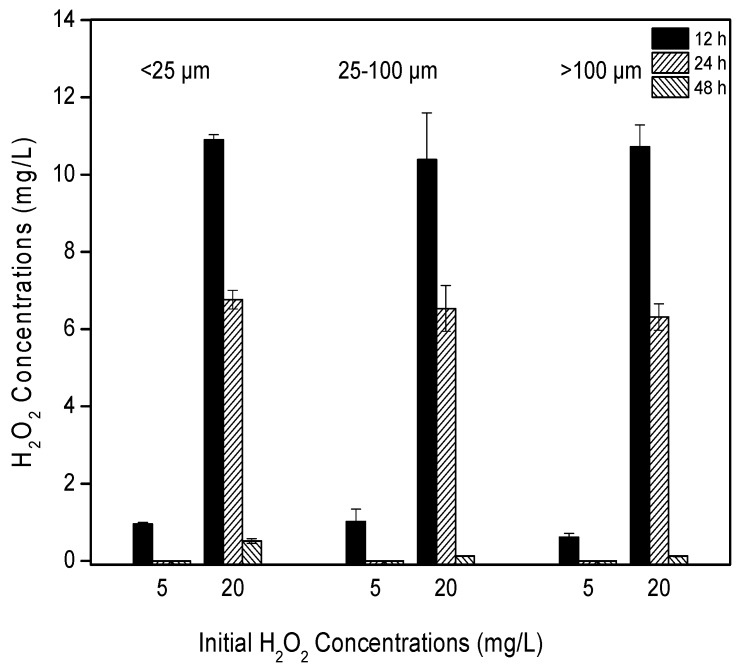
The residual concentration of hydrogen peroxide in flasks after 12, 24 and 48 h treatment with 5 and 20 mg/L H_2_O_2_. Values are represented as mean ± SD, *n* = 3.

The decay of H_2_O_2_ in the culture media were time- and concentration-dependent (Figure 1). The initial H_2_O_2_ concentration of 5 mg/L degraded to less than 1 mg/L within 12 h and was undetectable at 24 h. In contrast, the H_2_O_2_ concentration of 20 mg/L had a half-life of 12 h and was below detection limits in 48 h. The decay of H_2_O_2_ did not significantly differ among the different colony sizes (*p* = 0.26, Table 1).

### 2.2. Chlorophyll-a Content

**Figure 2:**
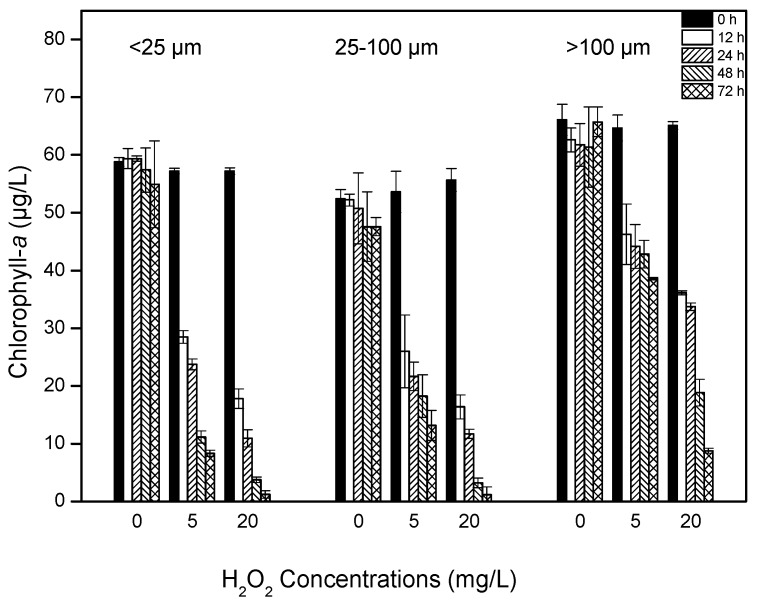
The contents of chlorophyll-a of algal colonies after 0, 12, 24, 48 and 72 h treatment with 0, 5 and 20 mg/L H_2_O_2_. Values are represented as mean ± SD, *n* = 3.

Hydrogen peroxide application at concentrations of 5 and 20 mg/L significantly lowered the amount of cyanobacterial chlorophyll-a after 12 h (Figure 2, Table 1). The initial chlorophyll-a concentration was approximately 60 μg/L for all the treatments. Chlorophyll-a content of the largest size class (>100 μm) decreased slowly after treatment with 5 mg/L H_2_O_2_ and dropped to approximately 38.6 μg/L (58% of the control level) after 72 h exposure. For the intermediate (25–100 μm) and smallest size class (<25 μm), chlorophyll-a content was reduced to 13.2 and 8.3 μg/L (26% and 15% of the control level), respectively, after 72 h of exposure. Under the 20 mg/L H_2_O_2_ condition, chlorophyll-a content decreased to 2.2%, 2.3% and 13% of the control level in the <25 μm, 25–100 μm and >100 μm classes, respectively, by the end of experiment. As a result of repeated-measures analysis of variance (ANOVA), the difference of chlorophyll-a content was highly significant among the H_2_O_2_ concentration group (*p* < 0.01) and colony size group (*p* < 0.01), respectively (Table 1). The results suggest that H_2_O_2_ was most effective at the highest concentration and in the smallest colony size class.

### 2.3. Effects of H_2_O_2_ on Photosynthetic Activity

**Figure 3:**
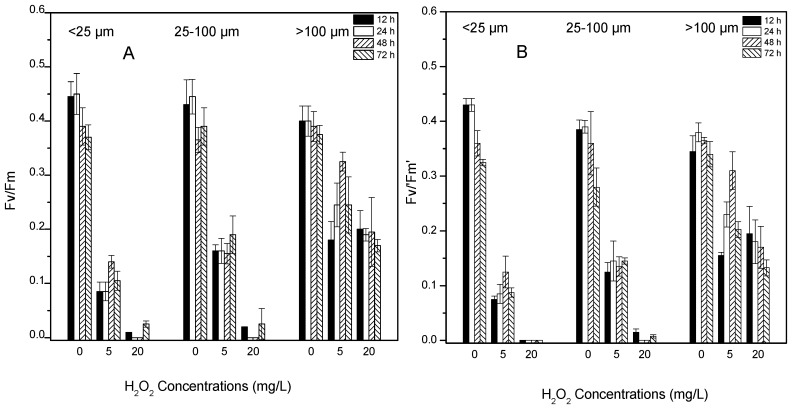
The value of maximum quantum yield Fv/Fm (**A**) and effective quantum yield Fv’/Fm’ (**B**) of algal colonies after 12, 24, 48 and 72 h treatment with 0, 5 and 20 mg/L H_2_O_2_. Values are represented as mean ± SD, *n* = 3.

The values of maximal quantum yield (Fv/Fm) and effective quantum yield (Fv’/Fm’) presented similar patterns after treatment with H_2_O_2_ (Figure 3A,B). The values were significantly lower at higher H_2_O_2_ concentrations and smaller colony sizes (*p* < 0.01) (Table 1). After treatment with 5 mg/L H_2_O_2_, Fv/Fm and Fv’/Fm’ both decreased after 12 h but showed clear increases thereafter. However, after treatment with 20 mg/L H_2_O_2_, Fv/Fm and Fv’/Fm’ values were drastically reduced in the small and intermediate size classes (<25 μm and 25–100 μm), being less than 5% of the control levels after 12 h of exposure. Photosynthetic activity appeared to be completely lost and exhibited only a slight recovery at 72 h. The large size class of *Microcystis* colonies (>100 μm) showed less inhibition, with more than 40% of photosynthetic activity remaining after treatment with 20 mg/L H_2_O_2_.

### 2.4. Effects of H_2_O_2_ on Esterase Activity

**Figure 4:**
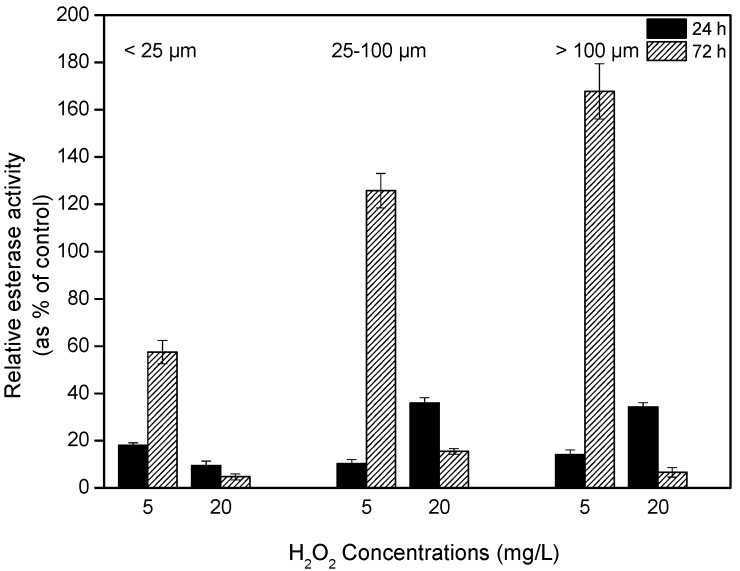
The relative esterase activity of algal colonies after 24 and 72 h treatment with 0, 5 and 20 mg/L H_2_O_2_. Values are represented as mean ± SD, *n* = 3.

When the *Microcystis* colonies were exposed to 5 mg/L H_2_O_2_, the esterase activities decreased to approximately 15% the activity of the controls after 24 h but increased thereafter, reaching 56, 125 and 167% of the control level for the small, intermediate and large size classes, respectively. However, at 20 mg/L H_2_O_2_, the esterase activity of *Microcystis* showed a continuous decline, implying that a high H_2_O_2_ concentration could completely destroy the viability of the cells (Figure 4).

### 2.5. Antioxidant Responses

#### 2.5.1. SOD and CAT Activities

**Figure 5:**
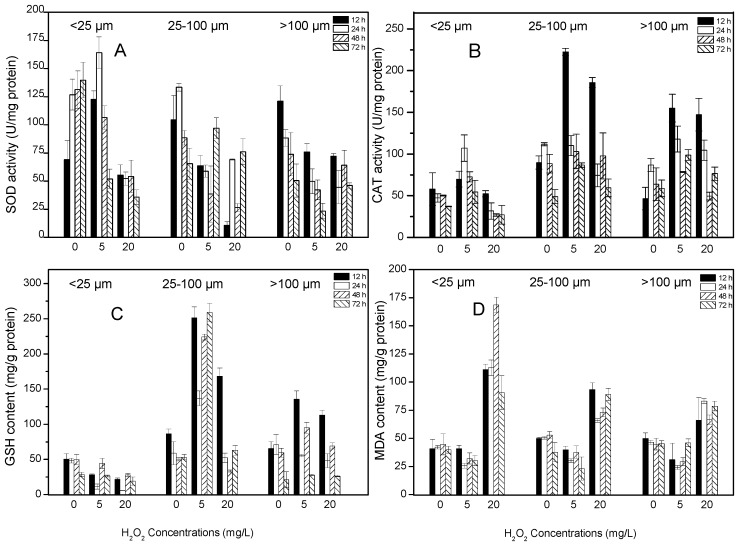
The activities of superoxide dismutase (SOD) (**A**), catalase (CAT) (**B**) and the contents of glutathione (GSH) (**C**), malondialdehyde (MDA) (**D**) after 12, 24, 48 and 72 h treatment with 0, 5 and 20 mg/L H_2_O_2_. Values are represented as mean ± SD, *n* = 3.

Initially, treatment with H_2_O_2_ generally stimulated SOD activity. In the smallest size class, SOD activity increased before 24 h, and decreased sharply with increasing incubation time at 5 mg/L H_2_O_2_ treatment, but SOD fluctuated at 20 mg/L H_2_O_2_ treatment. For the intermediate size class, SOD activity decreased before 48 h but then increased, becoming higher than that of the controls at 72 h. For the largest size class, SOD activity was initially inhibited and decreased to approximately 60% of the control activity at both H_2_O_2_ concentrations; however, at 20 mg/L H_2_O_2_, SOD recovered after 48 h (Figure 5A). In comparison with the pattern of SOD activity, the pattern of CAT activity was different. Inhibition of CAT activity was only observed in colonies of the small size class (<25 μm) at 20 mg/L H_2_O_2_. The activities of CAT were generally stimulated by H_2_O_2_ treatment, particularly in the middle and large size class, where CAT reached more than 2-fold of the control level at 12 h. However, CAT activity later showed a rapid decline in most treatments (Figure 5B).

#### 2.5.2. GSH and MDA Contents

In colonies <25 μm, GSH content decreased in both the 5 and 20 mg/L H_2_O_2_ treatments (*p* < 0.01) (Table 1). In contrast, in colonies 25–100 μm, GSH content was greatly enhanced in the 5 mg/L treatment, reaching levels 4.8-fold higher than those of the controls after 72 h treatment. At 20 mg/L H_2_O_2_, the GSH content showed a similar pattern between the intermediate and large size classes, increasing after 12 h and decreasing thereafter (Figure 5C). There was a significant difference in GSH content among the three size classes after treatment with H_2_O_2_ (*p* < 0.01) (Table 1). The contents of MDA varied with H_2_O_2_ concentration. At 5 mg/L H_2_O_2_, MDA content was lower than or similar to the control values at all colony size classes and all sampling times. In contrast, at 20 mg/L H_2_O_2_, MDA content generally increased and was highest in the small size class (Figure 5D).

### 2.6. Microcystin Concentrations

**Figure 6:**
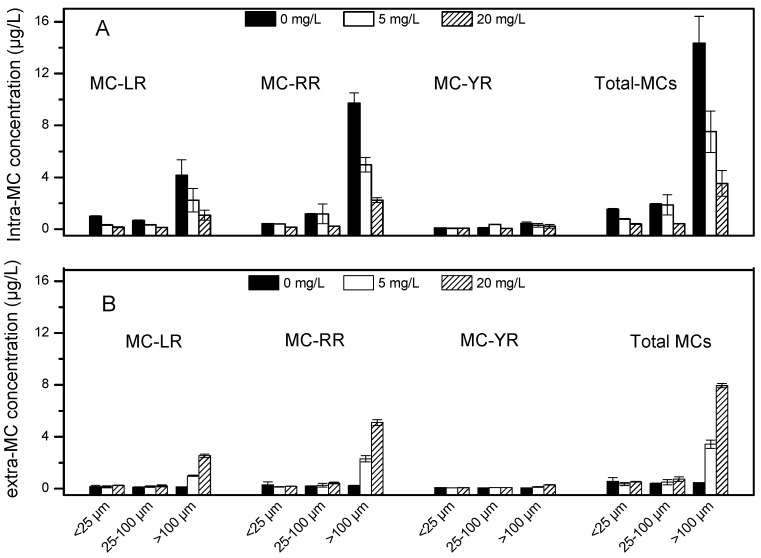
The concentration of intracellular-microcystins (MCs) (**A**) and extracellular-MCs (**B**) of MC-LR, MC-RR, MC-YR and total MCs after 24 h treatment with H_2_O_2_. Values are represented as mean ± SD, *n* = 3.

Total intracellular-MCs concentration was highest in the large size fraction, being 14.3 µg/L (Figure 6A). In contrast, total extracellular-MCs concentration was similarly low among all size class of *Microcystis* colonies, being approximately 0.5 µg/L (Figure 6B). The major contributors for MCs were MC-LR and MC-RR, while MC-YR was the minority for MCs. After the algae colonies were exposed to H_2_O_2_, intracellular-MCs concentrations decreased and extracellular-MCs concentrations increased, particularly in the large size fraction (Figure 6A,B). The concentration of extracellular- MCs was significantly higher in the large size class (>100 μm) (Table 2), with only a slight increase observed for the intermediate colonies (25–100 μm). In contrast, no significant increase of extracellular-MCs concentration was observed for the small colonies (<25 μm), after the addition of H_2_O_2_ (Figure 6B). This shift from intracellular to extracellular presence of the toxin is presumably the result of the release of toxins due to cell lysis.

## 3. Discussion

Although the degradation rate of H_2_O_2_ can increase with increasing algal density [23], our results indicated that H_2_O_2_ concentrations returned to background levels within 48 h regardless of the initial H_2_O_2_ concentration or colony size. Hydrogen peroxide has been reported to be an effective algaecide and a good option for the removal of cyanobacterial blooms. For example, in a previous study, the cyanobacterial population collapsed within a few days following treatment, whereas the remaining plankton community appeared much less affected [17]. Cyanobacteria are affected by H_2_O_2_ at concentrations 10 times lower than those that affect green algae and diatoms; i.e., the inhibitory effect of H_2_O_2_ is especially pronounced for cyanobacteria, showing that hydrogen peroxide is a compound selective to cyanobacteria [10]. A key issue in the treatment of harmful algal blooms with H_2_O_2_ is the selection of a suitable dosage. Previous studies have indicated that the appropriate dosage of H_2_O_2_ for the efficient inhibition of cyanobacteria can vary widely. For example, a bloom of the freshwater cyanobacterium *Planktothrix agardhii* was selectively removed with a low dosage of only 2 mg/L of H_2_O_2_ [17], 5 mg/L H_2_O_2_ was able to permanently inhibit the growth of benthic cyanobacteria under low light and low temperature conditions [24], and 10.2 mg/L H_2_O_2_ caused 85% of unicellular *Microcystis* to lose membrane integrity after 2 days of treatment [25]. Our results indicate that the effects of H_2_O_2_ on cyanobacteria depend on both colony size and H_2_O_2_ dosage. *Microcystis* colonies suffered cell lysis and photosynthetic inhibition under all treatments, although large colonies were less sensitive to H_2_O_2_ oxidative stress than were the smaller colonies. When the *Microcystis* colonies were exposed to 5 mg/L H_2_O_2_, photosynthetic activity partially recovered after 48 h, and relative esterase activity, following an initial sharp decline, increased by the end of the experiment. Esterase activity has been recognized as a useful marker for the detection of early responses to toxicants. As the toxic effect increases, the enzyme activity is reduced, resulting to a decline of the metabolic activity of algal cells. These findings suggest that this dosage is too low to completely suppress *Microcysti*s colonies. The dosage of 20 mg/L appeared to cause more severe damage to the photosynthetic activity of *Microcystis* colonies, particularly for those <100 µm in size, and it was able to fully destroy the metabolic activity of *Microcystis* colonies at all colony sizes. Colony formation has been reported as a strategy to reduce the damage from adverse factors, including zooplankton predation, phosphate-limited, resist to toxicant, etc. [14,26,27]. It is likely that smaller colonies possess thinner boundary layers, shallower required depth of penetration or relatively greater surface area of exposure to environmental stressors than do larger colonies and that as such, H_2_O_2_ can diffuse more readily into small colonies, causing damage. In addition, large colonies contain high contents of extracellular polymeric substances (EPS), and EPS has some buffering capacity against the algaecidal effect of H_2_O_2_ on cyanobacterial cells [14,28,29]. In the natural lake ecosystem, *Microcystis* colony sizes range from several micrometers to several hundred micrometers; however, the majority of colonies are less than 200 µm. Large-sized colonies dominated in July and August, small colonies prevailed before and after July and August, and intermediate colonies were observed throughout the year in lake Chaohu, where *Microcystis* colonies dominate during warm seasons [15]. Thus, the optimal dosage for efficiently suppressing cyanobacterial blooms would vary throughout the year. We recommend that H_2_O_2_ be applied early in the cyanobacterial bloom period, when small-sized colonies prevail. The application of H_2_O_2_ in low concentrations is expected to have fewer negative effects on the surrounding environment and water ecosystems compared with the applications of higher dosages.

Antioxidant responses are important defense mechanisms adopted by algae to scavenge reactive oxygen species (ROS), and these responses can alleviate the oxidative damage caused by environmental stress [16,18,30]. To alleviate oxidative damage, the antioxidant defense system is involved in scavenging excess ROS. Higher activities of SOD and CAT indicate a higher capacity for scavenging ROS. SOD is typically regarded as the first line of defense against the potential toxicity of ROS [16]. In the small colonies of the present study, SOD activity increased in response to oxidative stress and then declined after H_2_O_2_ decayed. In contrast, in the intermediate- and large-sized colonies, SOD activity decreased after initial exposure to H_2_O_2_ and then increased; an exception to this pattern was observed for the large colonies at 5 mg/L H_2_O_2_. These findings imply that H_2_O_2_ caused little oxidative stress on those two size classes colonies and that relatively high H_2_O_2_ concentrations can inhibit SOD activity. In contrast, CAT appeared to play an important role in the defense against oxidative stress in the intermediate- and large-sized *Microcystis* colonies, as CAT activity dramatically increased when the *Microcystis* colonies were exposed to H_2_O_2_. Reduced GSH is a crucial non-enzymatic antioxidant in the ascorbate glutathione cycle (AGC), and can remove ROS, such as hydroxide radical, lipid and alkyl peroxide [31]. Increased GSH has been observed in benthic cyanobacterial cells under H_2_O_2_ stress, which might reflect a strategy to protect algal cells from oxidative damage caused by low concentrations of H_2_O_2_ [24]. However, this protective action will be exhausted as exposure time and exposure dose increases. In the present study, GSH content increased sharply in the intermediate-sized colonies under the 5 mg/L H_2_O_2_ treatment. An increase was also observed in the large-size colonies under both concentrations of H_2_O_2_. In contrast, GSH declined in the small colonies following H_2_O_2_ treatment.

In the present study, parameters related to the antioxidant system exhibited different responses to H_2_O_2_ oxidative stress among the various colony size classes. This finding implies that *Microcystis* colonies of different sizes may have different strategies to remove the ROS caused by H_2_O_2_. For those colonies larger than 25 μm, the antioxidant enzyme CAT and the antioxidant substance GSH played major roles in responding to H_2_O_2_ stress. In the small colonies, SOD activity was readily stimulated and operated along with CAT to eliminate ROS. At 20 mg/L H_2_O_2_, the increased activities of SOD and CAT and the increased GSH content appeared insufficient for fully scavenging excessive ROS, as evidenced by strong inhibition of photosynthesis. When excess ROS are not effectively scavenged by the antioxidant system, the ROS radicals attack the unsaturated fatty acids located in cell membranes, causing lipid peroxidation and MDA accumulation [19]. Therefore, increased MDA content is a vital sign of cellular oxidative damage. A dramatic increase in MDA content was only observed under the 20 mg/L H_2_O_2_ treatment, indicating that the algae cells experienced oxidative damage. Furthermore, at this concentration, the extent of damage increased with decreasing colony size. In contrast, at 5 mg/L H_2_O_2_, MDA content remained at control levels in all colony size classes. It is possible that the high activity of the radical scavenging system inhibited the lipid peroxidation reaction and thus lowered MDA content.

An additional concern regarding the application of algaecides is MC release into the water following cell lysis, as MCs can cause serious health and environmental problems. Previous studies have shown that chemical algaecides can cause the release of intracellular-MCs [20,21]. Large *Microcystis* colonies (>100 μm) have been reported to have relatively high MC production and a high proportion of MC-producing genotypes [32]. In addition, MC-producing strains are more tolerant to H_2_O_2_ than are non-MC-producing genotypes since the oxidation of MCs competes with the algae colony for OH radicals [9,33,34]. In this study, the initial intracellular-MCs concentration in colonies >100 μm was significantly higher than the concentration in colonies <100 μm. Our results showed that the concentration of extracellular-MCs (in water) significantly increased with the increase in algaecide dosage, whereas the concentration of intracellular-MCs (in cells) decreased. The MC concentration in water reached 8 µg/L for colonies larger than 100 μm when chlorophyll-a concentration was around 60 µg/L, which exceeds the provisional guideline of 1 µg/L set by the World Health Organization (WHO). For the intermediate- and small-sized colonies, extracellular-MCs concentration was generally lower than this threshold after treatment with H_2_O_2_. Microcystin is reported as a potent liver tumor promoter. Although humans do not directly generally consume cyanobacteria, they may be regularly exposed to sub-lethal dosages of extracellular-MCs in drinking or recreational water derived from cyanobacteria-contaminated lakes and reservoirs. Moreover, extracellular-MCs have been demonstrated to be difficult to remove by traditional water treatments. Thus, the application of H_2_O_2_ as an algaecide should be performed at the early stages of a cyanobacterial bloom, when the cell densities are low and the *Microcystis* colonies are small, to limit the release of intracellular metabolites [25].

## 4. Conclusions

The optimal dosage of H_2_O_2_ required to efficiently eliminate *Microcystis* blooms depends on the colony size when algal biomass is same. *Microcystis* colonies less than 25 μm collapsed under treatment with 5 mg/L H_2_O_2_. A dosage of 20 mg/L H_2_O_2_ is needed to efficiently control *Microcystis* blooms in which the colonies are larger than 25 μm. The antioxidant responses of *Microcystis* varied with colony size. In colonies larger than 25 μm, the antioxidant enzyme CAT and antioxidant substance GSH played major roles at H_2_O_2_ concentrations below 20 mg/L. In small colonies, SOD activity was readily stimulated and functioned along with CAT activity to eliminate ROS. Chemical algaecides can lead to the release of intracellular-MCs. Following H_2_O_2_ treatment, the extracellular-MCs concentrations were relatively high when colonies were bigger than 100 μm. We recommend that H_2_O_2_ be applied early in the *Microcystis* bloom period, when small-sized colonies prevail. The application of H_2_O_2_ in low concentrations is expected to have fewer negative effects on the surrounding environment compared with applications of higher dosages. Meanwhile, H_2_O_2_ treatment can limit microcystin release in small colonies.

## 5. Materials and Methods

### 5.1. Sampling

Sampling was performed in July 2016 in Meiliang Bay of Lake Taihu, which is the third largest freshwater lake in China (surface area 2338 km^2^). It is a severely eutrophic lake with frequent cyanobacterial blooms, and long-term monitoring data have demonstrated that *Microcystis* is the dominant algae taxon during the summer blooms in Lake Taihu [35,36]. Microscopy observation revealed that *Microcystis*, including *M. aeruginosa, M. wesenbergii* and *M. flos-aquae*, constituted more than 95% of the phytoplankton in our sample. Natural *Microcystis* colonies were collected from the surface of the lake and stored with lake water in black bags to avoid exposure to high irradiance during transportation to the laboratory.

### 5.2. Experimental Procedures

Mesh sieves of 100 µm and 25 µm were used to fractionate the collected *Microcystis* samples. Samples were first filtered through a 100 μm mesh sieve, and those passing in the filtrate were further filtered through a 25 μm mesh sieve. The fractionated phytoplankton were then collected and re-suspended in filtered lake water (Whatman GF/C). During filtration, the mesh sieves were frequently back flushed with distilled water to prevent large colonies from becoming trapped. The resulting colony size classes were >100 μm, 25–100 μm and <25 μm. Microscopy observation revealed that the small colonies (<25 µm) were composed of unicellular *Microcystis* or colonies containing fewer than 20 cells. The intermediate size class (25 µm to 100 µm) contained colonies with dozens to hundreds of cells. The largest size class comprised colonies that could not pass through the 100 µm mesh sieve.

*Microcystis* colonies belonging to different size classes were placed into 27 flasks (nine flasks per colony size class) and diluted with 700 mL filtered lake water (Whatman GF/C) to ensure the final concentration of chlorophyll-a was the same among the flasks. Hydrogen peroxide was added to the flasks to a concentration of 0, 5, or 20 mg/L, with three replicates per concentration. The samples were then incubated under a 12 h (light)/12 h (dark) cycle with a light density of 2000–2500 L× at 25 ± 1 °C in an incubator. The flasks without H_2_O_2_ addition were sampled after 0, 12, 24, 48 and 72 h of incubation, which were used as the controls for H_2_O_2_ treatment at each sampling point.

### 5.3. Analytical Methods

#### 5.3.1. Measurement of H_2_O_2_

Water samples of 5 mL were collected from each flask after 12, 24 and 48 h of incubation, respectively. The concentrations of H_2_O_2_ were determined by a novel nonenzymatic colorimetric method [37]. *P*-nitrophenylboronic acid chemoselectively react with hydrogen peroxide under alkaline conditions to produce yellow p-nitrophenolate. The collected sample was centrifuged at 10,000 rpm for 5 min at 4 °C, and 1 mL of H_2_O_2_-containing supernatant was completely mixed with an equal volume of 2 mM *p*-nitrophenylboronic acid (*p*-NPBA) (pH 9, 150 mM bicarbonate-carbonate buffer). After 20 min, the absorbance of the colored product *p*-nitrophenol was recorded at 405 nm via spectrophotometer, and H_2_O_2_ concentration was calculated based on the standard calibration curve of H_2_O_2_.

#### 5.3.2. Measurement of Chlorophyll-a

In this study, the concentrations of chlorophyll-a in water samples were used as indicators of algal biomass [38]. In this experiment, water samples of 20 mL, collected from each flask after 0, 12, 24, 48 and 72 h of incubation, respectively, were filtered through GF/C glass microfiber filters (Whatman, Buckinghamshire, UK) for the determination of chlorophyll-a. The membranes containing the algae were ground into a homogenate with 10 mL 90% acetone, and then kept in the dark at 4 °C for 12 h. After centrifugation at 6000 rpm for 10 min, the supernatant was used to determine the contents of chlorophyll-a with a fluorescence spectrophotometer (RF-5301PC, Shimadzu Corporation, Kyoto, Japan) at a scan speed of 60 nm/min, band pass set of 5 nm, response time of 2 s and low PM gain. A synchronous scan with a wavelength difference Δλ = 258 nm was conducted from 608 nm to 708 nm at an excitation wavelength of 350 nm, and the maximum fluorescence peak was obtained at 670 nm. All standard pigments were purchased from Sigma (St. Louis, MO, USA) [38,39].

#### 5.3.3. Photosynthesis Measurements

Photosynthetic efficiency was determined by measuring the variable chlorophyll fluorescence of photosystem II (PSII) with a phyto-PAM (Walz, Effeltrich, Germany) equipped with a special Emitter-Detector Unit Phyto-ED using the PHYTO-PAM software Phyto-WIN v2.13. Water samples of 5 mL, collected after 12, 24, 48 and 72 h of incubation, respectively, were added to 25 μL ascorbic acid to terminate the oxidation of H_2_O_2_ and to eliminate the remaining H_2_O_2_. First, after 15 min dark-adapted, F_0_ was determined as the fluorescence of cells stimulated by a weak probe light immediately after the acclimatization period. Second, the maximum fluorescence signal Fm was measured by a 600 ms pulse of saturating irradiance. Third, the actinic light was turned on, and these dark-adaptation samples transitioned to a light-adapted state. Next, in the light adapted state, the maximum fluorescence signal Fm’ was determined by a 600 ms pulse of saturating irradiance. F_0_’ was the current instantaneous fluorescence signal in the light-adapted steady-state. The maximum quantum yield (Fv/Fm) and effective quantum yield (Fv’/Fm’) of PS II were calculated as Fv/Fm = (Fm − F_0_)/Fm and Fv’/Fm’ = (Fm’ − F_0_’)/Fm’, respectively [40,41].

#### 5.3.4. Esterase Activity Measurement

Esterase activity was detected according to Delphine’s methods with minor modification [42]. Water samples of 5 mL, collected after 0, 24 and 72 h of incubation, respectively, were added to 12.5 μL ascorbic acid and then scattered by low-power ultrasonic vibration (XO-1000D, 20 kHz, Nanjing Xianou Instruments Manufacture Co., Ltd, Najing, China) for <1 min. Then, 1 mL of each sample was passed through a 48 µm pore-size sieve to eliminate large particles and avoid blocking the nozzle. The filtrate was then stained with 25 μmol/L FDA (Sigma F-7378) in the dark for 8 min [43]. All analyses were performed with a FACSJazz^SE^ flow cytometer (Becton Dickinson, San José, CA, USA) equipped with an FL1 detector.

#### 5.3.5. Array of Antioxidant Responses

Water samples of 20 mL, collected after 12, 24, 48 and 72 h of incubation, respectively, were filtered with 1.2 µm pore size GF/C glass microfiber filters (Whatman, Buckinghamshire, UK). The membrane and its contents were wet grinded with 4 mL phosphate buffer solution (0.1 mol/L, pH = 7.0) and homogenized on ice by low-power ultrasonic vibration (20 kHz) for 2 min (ultrasonic vibration time: 4 s, rest time: 4 s). Extracts were centrifuged for 10 min at 8000 rpm at 4 °C. The supernatant was used for the following array, which included total protein content, non-enzymatic antioxidant contents, and antioxidant enzyme activities. These were determined spectrophotometrically by using an assay kit (Jiancheng Biotech, Nanjing, China) following the manufacturer’s instructions [19].

SOD activity was determined by the nitro blue tetrazolium (NBT) method [44]. One unit of SOD activity was defined as the quantity of SOD required to produce a 50% reduction of NBT/mg protein in 1 mL reaction mixtures. CAT activity was measured, following the method of Goth [45], by monitoring the decrease in absorbance at 240 nm as a consequence of H_2_O_2_ consumption. One unit of CAT activity was defined as the amount of enzyme that degraded 1 μmol H_2_O_2_ per minute at 37 °C [30].

MDA content was measured according to method [46], which is based on the chromogenic reaction of MDA with thiobarbituric acid. GSH content was determined by the 5,5’-dithiobis-(2-nitrobenzoic acid)-glutathione reductase (DTNB-GR) recycling assay according to the method of Anderson. Total soluble protein (TSP) content was determined by the Coomassie blue-dye binding assay with Bradford’s method [47]. A standard curve of protein quantity was obtained by using bovine serum albumin (Sigma, St. Louis, MO, USA). The activities of the antioxidant enzymes and the contents of the non-enzymatic antioxidants in the algae cells were expressed in units per mg of protein (U/mg protein) and mg/g protein, respectively.

#### 5.3.6. Microcystin Analysis

Water samples of 80 mL were collected from each flask after 24 h of incubation and filtered through GF/C glass microfiber filters (pore size, 1.2 µm; Whatman, Buckinghamshire, UK). The membranes were collected to analyze the intracellular-MCs and the filtrate was collected to analyze extracellular-MCs, respectively. For intracellular-MCs extraction, the filters and their contents were ground with 1 mL 5% aqueous acetic acid using a Fast Prep-24 automated homogenizer (MP Biomedicals, Santa Ana, CA, USA) with 0.5 mm silica beads. Then, intracellular-MCs were extracted by using 20 mL 80% aqueous methanol for 30 min with shaking. After centrifugation (9500 rpm, 10 min), the supernatant was diluted 1:5 with distilled water. The diluted supernatant was then concentrated using a solid-phase extraction cartridge (C18, 0.5 g) that had been rinsed with 10 mL distilled water and 10 mL 20% methanol. The 10 mL of elute obtained from the cartridge by using (0.1% TFA) methanol was blown to dryness with nitrogen at 40 °C. The resulting residues were all dissolved in 1 mL 100% methanol and were transferred into a small brown bottle, then blown dry with nitrogen. Then, the residues were dissolved in 200 µL of 50% aqueous methanol before HPLC analysis. For extracellular-MCs extraction, 80 mL filtrate was directly concentrated by using a solid phase extraction cartridge (C18, 0.5 g). The subsequent steps of blow-drying with nitrogen and dissolving in methanol were performed as described for the intracellular-MCs extraction [7].

The extracted intracellular-MCs and extracellular-MCs were analyzed using a high performance liquid chromatography (HPLC) system equipped with an ODS column (Agilent EclipseXDB-C18, 5 µm, 4.6 × 150 mm) and a photodiode array detector (Agilent 1200, Agilent, Palo Alto, CA, USA). Mobile phases were Milli-Q water and acetonitrile, both containing 0.05% (*v*/*v*) trifluoroacetic acid (TFA). Chromatographic separation was achieved at a flow rate of 1 mL/min using a gradient starting at 30% aqueous acetonitrile increasing to 35% over the next 10 min followed by an increase to 70% over the next 30 min. Microcystin concentration was quantified based on their retention time and characteristic UV spectra. The standards of MC-RR, MC-YR, and MC-LR were purchased from Sigma (München, Germany). The order of the three peaks was MC-RR, MC-YR, and MC-LR, and the retention time was 5.79, 10.05, and 11.19 min, respectively. The maximum absorption peak was at 239 nm. Total MC concentration was quantified as the sum of all MCs peaks.

#### 5.3.7. Statistical Analysis

Repeated measures ANOVA were conducted to identify significant differences over time; the Mauchley’s test was first tested. The treatment of different colony size with different concentrations of H_2_O_2_ on the concentration of MCs has been analyzed by a two-way ANOVA. The normality and variance homogeneity test of data were first tested, and then multiple-comparison tests of least significance difference (LSD) were used as a post hoc procedure to evaluate which treatment groups significantly differed from each other. All of the data analyses were performed using SPSS 16.0 (SPSS Inc., Chicago, IL, USA) and Origin 8.5 (OriginLab Corporation, Northampton, MA, USA) for Windows.

## Figures and Tables

**Table 1:** Repeated-measures analysis of variance (ANOVA) for different variables over time within 0, 5 and 20 mg/L H_2_O_2_ treatments for three size classes colonies (<25 μm, 25–100 μm and >100 μm).

Variable	H_2_O_2_		Chlorophyll-a		Fv/Fm		Fv’/Fm’		FDA	
Effects	*F*	*p*-Value	*F*	*p*-Value	*F*	*p*-Value	*F*	*p*-Value	*F*	*p*-Value
Time	8832.23	<0.01 *	638.07	<0.01*	0.98	0.37	21.79	<0.01 *	274.71	<0.01 *
Time * Concentration	4492.83	<0.01 *	149.05	<0.01*	16.33	<0.01 *	21.16	<0.01 *	110.02	<0.01 *
Time × Size	0.47	0.83	5.63	<0.01*	6.26	<0.01 *	5.19	<0.01 *	23.06	<0.01 *
Concentration	9942.51	<0.01 *	2562.62	<0.01*	987.99	<0.01 *	953.76	<0.01 *	267.84	<0.01 *
Size	1.42	0.26	617.68	<0.01*	96.23	<0.01 *	98.72	<0.01 *	21.23	<0.01 *
Concentration × Size	1.10	0.38	45.06	<0.01*	42.08	<0.01 *	40.36	<0.01 *	17.91	<0.01 *
Variable	SOD		CAT		GSH		MDA			
Effects	*F*	*p*-Value	*F*	*p*-Value	*F*	*p*-Value	*F*	*p*-Value		
Time	17.97	<0.01 *	114.99	<0.01 *	102.51	<0.01 *	71.91	<0.01*		
Time × Concentration	5.67	<0.01 *	25.73	<0.01 *	24.37	<0.01 *	8.90	<0.01*		
Time × Size	21.96	<0.01 *	28.88	<0.01 *	40.28	<0.01 *	29.88	<0.01*		
Concentration	170.31	<0.01 *	113.85	<0.01 *	225.26	<0.01 *	330.40	<0.01*		
Size	70.11	<0.01 *	196.84	<0.01 *	469.17	<0.01 *	160.14	<0.01*		
Concentration × Size	31.39	<0.01 *	11.45	<0.01 *	179.12	<0.01 *	44.76	<0.01*		

* Statistically significant when compared among groups.

**Table 2:** Two-way ANOVA for the concentration of MCs among colony size and H_2_O_2_ concentrations. Different letters indicate a significant difference among groups (*p* < 0.05).

Variable	H_2_O_2_ Concentration			Colony Size		
	0 mg/L	5 mg/L	20 mg/L	<25 μm	25–100 μm	>100 μm
Intracellular-MCLR	a	b	c	a	a	b
Intracellular-MCRR	a	b	c	a	b	c
Intracellular-MCYR	a	a	b	a	b	c
Intracellular-MCs	a	b	c	a	a	b
Extracellular-MCLR	a	b	c	a	a	b
Extracellular-MCRR	a	b	c	a	a	b
Extracellular-MCYR	a	b	c	a	a	b
Extracellular-MCs	a	b	c	a	a	b
